# Annual of the Universal Medical Sciences

**Published:** 1890-11

**Authors:** 


### Book Reviews.

Annual of the Universal Medical Sciences. A Yearly Report of the Progress of the General Sanitary Sciences throughout the World. Edited by Charles E. Sajous, M.D., and Seventy Associate Editors, Assisted by over Two Hundred Corresponding Editors, Collaborators and Correspondents. Illustrated with Chromo-Lithographs, Engravings and Maps. F. A. Davis, Philadelphia.

The issue of 1890 fully maintains the high standard of excellence of its predecessors in every particular, and excels them in many.

" The improvements in this year's issue mainly consist in the creation of departments on subjects heretofore considered under general heads. Syphilis, for instance, under the editorship of Prof. J. William White, of Philadelphia, appears as a special section, replete with information that could hardly otherwise be presented satisfactorily. Surgical Mycoses, edited by Prof. Ernest Laplace, of Philadelphia, is another subject so treated; while that of Thoracic Surgery, by Prof. J. McFadden Gaston, of Atlanta, forms a department, the value of which will become apparent."

Many other important additions and modifications have been made, most important of which are in Oral Surgery, under Dr. R. Matas, of New Orleans; Orthopædic Surgery, under Profs. Lewis A. and Reginald H. Sayre, of New York; and Bacteriology, under Dr. Harold C. Ernest, of Boston.

The general excellence of the *Annual* must be appreciated immediately upon perusal, and it justly merits its continued success.

